# Comparative Genomics of *Aeromonas hydrophila* Secretion Systems and Mutational Analysis of *hcp1* and *vgrG1* Genes From T6SS

**DOI:** 10.3389/fmicb.2018.03216

**Published:** 2019-01-09

**Authors:** Hasan C. Tekedar, Hossam Abdelhamed, Salih Kumru, Jochen Blom, Attila Karsi, Mark L. Lawrence

**Affiliations:** ^1^College of Veterinary Medicine, Mississippi State University, Starkville, MS, United States; ^2^Bioinformatics and Systems Biology, Justus-Liebig-University Giessen, Giessen, Germany

**Keywords:** *Aeromonas hydrophila*, comparative genomics, secretion systems, T6SS, Hcp, VgrG

## Abstract

Virulent *Aeromonas hydrophila* causes severe motile *Aeromonas* septicemia in warmwater fishes. In recent years, channel catfish farming in the U.S.A. and carp farming in China have been affected by virulent *A. hydrophila*, and genome comparisons revealed that these virulent *A. hydrophila* strains belong to the same clonal group. Bacterial secretion systems are often important virulence factors; in the current study, we investigated whether secretion systems contribute to the virulent phenotype of these strains. Thus, we conducted comparative secretion system analysis using 55 *A. hydrophila* genomes, including virulent *A. hydrophila* strains from U.S.A. and China. Interestingly, tight adherence (TaD) system is consistently encoded in all the vAh strains. The majority of U.S.A. isolates do not possess a complete type VI secretion system, but three core elements [*tssD* (*hcp*), *tssH*, and *tssI* (*vgrG*)] are encoded. On the other hand, Chinese isolates have a complete type VI secretion system operon. None of the virulent *A. hydrophila* isolates have a type III secretion system. Deletion of two genes encoding type VI secretion system proteins (*hcp1* and *vgrG1*) from virulent *A. hydrophila* isolate ML09-119 reduced virulence 2.24-fold in catfish fingerlings compared to the parent strain ML09-119. By determining the distribution of genes encoding secretion systems in *A. hydrophila* strains, our study clarifies which systems may contribute to core *A. hydrophila* functions and which may contribute to more specialized adaptations such as virulence. Our study also clarifies the role of type VI secretion system in *A. hydrophila* virulence.

## Introduction

*Aeromonas hydrophila* is common in freshwater environments and causes disease in fish, reptiles, amphibians, and humans (Janda and Abbott, 2010; Tomás, 2012). The U.S.A. and China aquaculture industries have had significant losses due to *A. hydrophila* disease (Nielsen et al., 2001). In the southeastern U.S.A., severe *A. hydrophila* outbreaks began impacting the catfish aquaculture industry in 2009 and are caused by a clonal group of strains named virulent *A. hydrophila* (vAh) (Hemstreet, 2010; Hossain et al., 2014).

Comparative genomics methods have helped identify taxonomically mislabeled *A. hydrophila* genomes in Genbank (Beaz-Hidalgo et al., 2015). The same methods also revealed that the U.S.A. vAh clonal lineage is similar to a clonal lineage of *A. hydrophila* that is responsible for significant economic losses in the Chinese aquaculture industry (Griffin et al., 2013; Hossain et al., 2013; Zhang et al., 2014; Pang et al., 2015). Both clonal groups are categorized as sequence type ST251 (Rasmussen-Ivey et al., 2016). It has been theorized that the U.S.A. vAh originated from transport of carrier fish from Asia (Hossain et al., 2014).

Comparative genomics has revealed that the vAh clonal group has unique characteristics. Some of the unique biochemical pathways include sialic acid biosynthesis, *myo-*inositol utilization, and L-fucose metabolism. They also have unique O-antigen biosynthesis and characteristic mobilome elements and secretion systems (Hossain et al., 2013; Pang et al., 2015). Intriguingly, Asian vAh isolates encode all the core components of type VI secretion system (T6SS), whereas most of the U.S.A. vAh isolates carry remnants of the T6SS (Rasmussen et al., 2016).

Several virulence mechanisms of *A. hydrophila* including secretion systems, motility, toxins, tissue-destructive enzymes, iron acquisition, and S-layer have been studied (Tomás, 2012). Secretion systems are used by bacteria to interact with the environment, including host adaptation and competing against other bacteria (Cianfanelli et al., 2016). A thorough investigation of secretion systems distribution in *A. hydrophila*, including vAh, other fish disease strains, and environmental strains, has not been conducted. Hence, in this study, we analyzed 55 *A. hydrophila* genomes from distinct geographical origins and hosts. We also evaluated type 4 pili (T4P), tight adherence systems (Tad), and flagella components due to their sequence similarity to secretion systems. Potential host-pathogen interactions of the identified secretion system proteins were evaluated.

In the current study, we conducted comparative genomics of secretion systems encoded by *A. hydrophila*. Our analysis showed that all the evaluated *A. hydrophila* genomes encode the whole operon or remnants of T6SS. To clarify the function of T6SS genes in vAh, we mutated two T6SS genes in strain ML09-119, and we determined the virulence of mutant strains in catfish fingerlings. Overall, the comparative genomics and mutational analyses reported here clarify the distribution of various secretion systems in *A. hydrophila* and provide functional information on the role of T6SS components in vAh.

## Materials and Methods

### Genome Sequences and Annotation

The genome sequences (including complete sequences, draft assemblies, and raw reads) of 55 *A. hydrophila* strains were downloaded from the National Center for Biotechnology Information (Table 1). Raw data were assembled using CLC workbench 6.5.1 after trimming sequence reads, followed by error correction and contig creation. All unannotated genomes were annotated by RAST (Brettin et al., 2015). All selected genomes had at least 95% average nucleotide identity (ANI).

**Table 1:** *A. hydrophila* genomes used in comparative genomic analyses.

**No**.	**Species**	**Strain**	**Location**	**Source**	**Level**	**Accession**	**References**
1	*A. hydrophila*	Arkansas 2010	USA	Catfish	Contig	NZ_LYZH00000000.1	Tekedar et al., 2017
2	*A. hydrophila*	ML09-119	USA	Catfish	Complete	NC_021290.1	Tekedar et al., 2013
3	*A. hydrophila*	ML09-122	USA	Catfish	Contig	NZ_LRRY00000000.1	Tekedar et al., 2016b
4	*A. hydrophila*	ML09-121	USA	Catfish	Contig	NZ_LRRX00000000.1	Tekedar et al., 2016b
5	*A. hydrophila*	AL10-121	USA	Catfish	Contig	NZ_LRRW00000000.1	Tekedar et al., 2016b
6	*A. hydrophila*	AL09-71	USA	Catfish	Complete	NZ_CP007566.1	Pridgeon et al., 2014b
7	*A. hydrophila*	pc104A	USA	Soil	Complete	NZ_CP007576.1	Pridgeon et al., 2014a
8	*A. hydrophila*	S14-296	USA	Catfish	Contig	SAMN05292365	Rasmussen-Ivey et al., 2016
9	*A. hydrophila*	S14-606	USA	Catfish	Contig	SAMN05292366	Rasmussen-Ivey et al., 2016
10	*A. hydrophila*	S13-612	USA	Catfish	Contig	SAMN05292362	Rasmussen-Ivey et al., 2016
11	*A. hydrophila*	S13-700	USA	Catfish	Contig	SAMN05292363	Rasmussen-Ivey et al., 2016
12	*A. hydrophila*	Ahy_Idx7_1	USA	Catfish	Contig	SAMN05292361	Rasmussen-Ivey et al., 2016
13	*A. hydrophila*	ALG15-098	USA	Catfish	Contig	SAMN05223361	Rasmussen-Ivey et al., 2016
14	*A. hydrophila*	IPRS-15-28	USA	Catfish	Contig	SAMN05223362	Rasmussen-Ivey et al., 2016
15	*A. hydrophila*	ML10-51K	USA	Catfish	Contig	SAMN05223363	Rasmussen-Ivey et al., 2016
16	*A. hydrophila*	S14-458	USA	Catfish	Contig	SAMN05223364	Rasmussen-Ivey et al., 2016
17	*A. hydrophila*	S15-130	USA	Catfish	Contig	SAMN05223365	Rasmussen-Ivey et al., 2016
18	*A. hydrophila*	S15-400	USA	Catfish	Contig	SAMN05223367	Rasmussen-Ivey et al., 2016
19	*A. hydrophila*	ZC1	USA	Grass carp	Contig	SAMN02404465	Hossain et al., 2014
20	*A. hydrophila*	AL09-79	USA	Catfish	Contig	NZ_LRRV00000000.1	Tekedar et al., 2016b
21	*A. hydrophila*	2JBN101	China	Crucian carp	Contig	NZ_LXME00000000.1	Zhang et al., 2013
22	*A. hydrophila*	D4	China	Wuchang bream	Complete	NZ_CP013965.1	Tran et al., 2015
23	*A. hydrophila*	JBN2301	China	Carp	Complete	NZ_CP013178.1	Yang et al., 2016
24	*A. hydrophila*	S15-591	USA	Catfish	Contig	SAMN05223368	Rasmussen-Ivey et al., 2016
25	*A. hydrophila*	J-1	China	Carp	Complete	NZ_CP006883.1	Pang et al., 2015
26	*A. hydrophila*	NJ-35	China	Carp	Complete	NZ_CP006870.1	Pang et al., 2015
27	*A. hydrophila*	GYK1	China	Chinese perch	Complete	NZ_CP016392.1	Pan et al., 2004
28	*A. hydrophila*	SNUFPC-A8	S. Korea	Salmon	Contig	NZ_AMQA00000000.1	Han et al., 2013
29	*A. hydrophila*	NF1	USA	Human clinical	Contig	NZ_JDWB00000000.1	Grim et al., 2014
30	*A. hydrophila*	Ae34	Sri Lanka	Carp	Contig	NZ_BAXY00000000.1	Jagoda et al., 2014
31	*A. hydrophila*	M052	Malaysia	Waterfall	Contig	NZ_MAKI00000000.1	N/A
32	*A. hydrophila*	M053	Malaysia	Waterfall	Contig	NZ_MAKJ00000000.1	N/A
33	*A. hydrophila*	M062	Malaysia	Waterfall	Contig	NZ_JSXE00000000.1	Chan et al., 2015
34	*A. hydrophila*	AHNIH1	USA	Human clinical	Complete	NZ_CP016380.1	Hughes et al., 2016
35	*A. hydrophila*	AL06-06	USA	Goldfish	Complete	NZ_CP010947.1	Tekedar et al., 2015
36	*A. hydrophila*	ATCC 7966	USA	Milk tin	Complete	NC_008570.1	Seshadri et al., 2006
37	*A. hydrophila*	AL97-91	USA	Tilapia	Contig	NZ_CM004591.1	Tekedar et al., 2017
38	*A. hydrophila*	MN98-04	USA	Tilapia	Contig	NZ_CM004592.1	Tekedar et al., 2017
39	*A. hydrophila*	AH-1	Canada	Moribund fish	Contig	NZ_LYXN00000000.1	Forn-Cuní et al., 2016
40	*A. hydrophila*	RB-AH	Malaysia	Soil	Contig	NZ_JPEH00000000.1	Emond-Rheault et al., 2015
41	*A. hydrophila*	NF2	USA	Human clinical	Contig	NZ_JDWC00000000.1	Grim et al., 2014
42	*A. hydrophila*	S14-230	USA	Catfish	Contig	SAMN05292364	Rasmussen-Ivey et al., 2016
43	*A. hydrophila*	48_AHYD	USA	Human clinical	Scaffold	NZ_JVFM00000000.1	Roach et al., 2015
44	*A. hydrophila*	53_AHYD	USA	Human clinical	Scaffold	NZ_JVDL00000000.1	Roach et al., 2015
45	*A. hydrophila*	56_AHYD	USA	Human clinical	Scaffold	NZ_JVCD00000000.1	Roach et al., 2015
46	*A. hydrophila*	52_AHYD	USA	Human clinical	Scaffold	NZ_JVDW00000000.1	Roach et al., 2015
47	*A. hydrophila*	50_AHYD	USA	Human clinical	Scaffold	NZ_JVES00000000.1	Roach et al., 2015
48	*A. hydrophila*	AH10	China	Grass carp	Complete	NZ_CP011100.1	Xu et al., 2013
49	*A. hydrophila*	TN-97-08	USA	Bluegill	Contig	NZ_LNUR00000000.1	Tekedar et al., 2016a
50	*A. hydrophila*	FDAARGOS_78	USA	Human stool	Contig	JTBD01000000	N/A
51	*A. hydrophila*	226	Malaysia	Human urine	Contig	NZ_JEML00000000.1	Chan et al., 2011
52	*A. hydrophila*	M013	Malaysia	Waterfall	Contig	NZ_JRWS00000000.1	Tan et al., 2015a
53	*A. hydrophila*	AD9	USA	Wetland sediment	Contig	NZ_JFJO00000000.1	Lenneman and Barney, 2014
54	*A. hydrophila*	M023	Malaysia	Waterfall	Contig	NZ_JSWA00000000.1	Tan et al., 2015b
55	*A. hydrophila*	Ranae CIP 107985	USA	Fish/Ranae	Scaffold	NZ_CDDC00000000.1	Colston et al., 2014

*N/A, Not available*.

### Phylogenetic Tree Creation

A phylogenetic tree was built from the complete core genomes of 55 *A. hydrophila* strains, which included 115,335 coding sequences (2,097/genome) with 101,851,090 amino acid residues (1,851,838/genome). The gene sets of the core genome were aligned one by one using MUSCLE (Edgar, 2004), and alignments were concatenated. This alignment was used to compute a Kimura distance matrix, which was used as input for the Neighbor-Joining algorithm as implemented in PHYLP (Felsenstein, 1989). The resulting tree was verified by bootstrapping with 250 iterations.

### ANI and AAI Calculation

Average nucleotide identity and average amino acid identity (AAI) values (Konstantinidis and Tiedje, 2005a,b; Konstantinidis et al., 2006) were calculated using EDGAR (Konstantinidis and Tiedje, 2005b). Briefly, the average amino acid identities were based on all protein sequences encoded by genes in the core genome (2,097 per genome). Percent identity values were extracted from BLASTP (Altschul et al., 1990) results that are stored in the EDGAR database, summed up, and averaged for every pair of genomes. ANI using BLAST (ANIb) was based on BLASTN results as described (Goris et al., 2007) using the same cutoffs as JSpeciesWS (Richter and Rossello-Mora, 2009).

### Identification of Secretion Systems

MacSyFinder was used with default features to identify secretion systems from the *A. hydrophila* genomes. The “unordered” type of dataset option was chosen because the majority of the evaluated genomes were draft genomes. The topology of the replicon was linear/circular, maximal *E*-value was 1.0, maximal independent *E*-value was 0.001, and minimal profile coverage was 0.5. Both mandatory genes and accessory genes were identified (Abby et al., 2014, 2016).

### Bacterial Strains and Plasmids

Bacterial strains and plasmids used in this study are listed in Table 2. *Aeromonas hydrophila* strain ML09-119 represents the vAh clonal group impacting U.S.A. channel catfish aquaculture. The strain was grown on brain heart infusion (BHI) agar or broth (Difco, Sparks, MD, USA) and incubated at 37°C. *Escherichia coli* strain CC118 λ*pir* was used for cloning, and strain BW19851 was used for transferring suicide plasmid pMEG-375 into *A. hydrophila* by conjugation. *Escherichia coli* strains were cultured in Luria–Bertani (LB) agar and broth (Difco) and incubated at 37°C. The following antibiotics and reagents (Sigma-Aldrich, Saint Louis, MN, USA) were used when needed: ampicillin (100 μg/ml), chloramphenicol (10–25 μg/ml), colistin (12.5 μg/ml), sucrose (5%), and mannitol (0.35%).

**Table 2:** Bacterial strains and plasmids used in the present study.

**Strain or plasmid**	**Description**	**References**
*A. hydrophila* ML09-119	Isolate from a disease outbreak on a commercial catfish farm	Griffin et al., 2013
*vAh*Δ*hcp1*	*A. hydrophila* ML09-119 derivative; Δ*hcp1*	This study
*vAh*Δ*vgrG1*	*A. hydrophila* ML09-119; Δ*vgrG*	This study
***E. coli***		
CC118λ*pir*	Δ(*ara*-*leu*); *araD*; Δ*lacX74*; *galE*; *galK*; *phoA20*; *thi-1*; *rpsE*; *rpoB*; *argE*(Am); *recAl*; λ*pir*R6K	Herrero et al., 1990
BW19851	*RP4-2 (Km::Tn7, Tc::Mu-1), DuidA3::pir+, recA1, endA1, thi-1, hsdR17, creC510*	Metcalf et al., 1994
**Plasmid**		
pMEG-375	8,142 bp, Amp^r^, Cm^r^, *lacZ*, R6K *ori, mob incP, sacR sacB*	Dozois et al., 2003
pAhΔ*hcp1*	10,173 bp, Δ*hcp1*, pMEG-375	This study
pAhΔ*vgrG1*	10,160 bp, Δ*vgrG1*, pMEG-375	This study

### In-frame Deletion of *A. hydrophila* Genes

Two chromosomal in-frame deletion mutants of type six secretion system (T6SS) effector genes *hcp1* (AHML_05970) and *vgrG1* (AHML_05975) were constructed by allelic exchange and homologous recombination using suicide plasmid pMEG-375 containing the counter-selectable marker *sacB* (Dozois et al., 2003). Recombinant DNA and mutant construction procedures were completed as described previously (Abdelhamed et al., 2013). Briefly, four primers (A, B, C, and D) were designed for each gene using Primer3 (Untergasser et al., 2012) (Table 3). Compatible restriction enzyme sites were embedded in A and D primers (bold line in primers A and D) for cloning, and the reverse complement of primer B was added to the 5′ end of primer C (underlined letters in primers C) to allow fusion of PCR fragments by overlap extension PCR (Horton et al., 1989). The upstream (fragment AB) and downstream (fragment CD) of each gene was amplified using two sets of primers. PCR fragments AB and CD were annealed at the overlapping regions and were amplified as a single fragment using primers A and D. The fusion products were purified, digested, ligated into digested pMEG-375, electroporated into *E. coli* CC118λ*pir*, and spread on LB agar plus ampicillin.

**Table 3:** Primers used to generate and verify in-frame deletion of vAh genes.

	**Primer ID**		**Sequence 5′-3′**	**RE**
*hcp1*	hcp1F01	A	AAA**TCTAGA**TCCTATGTGCCTGAGTGTGC	*Xba*I
	hcp1R1000	B	AATGACACTCGACCAAACCA	
	hcp1F1000	C	TGGTTTGGTCGAGTGTCATTGAGGCCTAACGCTCGATCT	
	hcp1R01	D	AAA**GAGCTC**AGGTCGGTTTCCCGGTACT	*Sac*I
	hcp1Seq		GCTGGCTCTCCATGCATATT	
*vgrG1*	vgrG1F01	A	AAA**TCTAGA**AAGGTAAAACCCAGGGCAAT	*Xba*I
	vgrG1R1000	B	TGTGCTGTCTGCCATGAAG	
	vgrG1F1000	C	CTTCATGGCAGACAGCACACGACTGATTGAGGTTTCCGTA	
	vgrG1R01	D	AAA**GAGCTC**CAGGCTGGTGTCTCGATTTT	*Sac*I
	vgrG1Seq		GCAAAGCACAACAGAGGCTA	

*Bold letters at the 5′ end of the primer sequence represent restriction enzymes (RE) site added. AAA nucleotides were added to the end of each primer containing a RE site. Underlined bases in primer C indicate reverse complemented primer B sequence*.

The resulting plasmids were purified from *E. coli* CC118λ*pir* and transferred into *A. hydrophila* ML09-119 by conjugation using *E. coli* BW19851. Transconjugants were selected on plates containing chloramphenicol and colistin; chloramphenicol was used to select the integration of pMEG-375 in *A. hydrophila* chromosome while colistin was used as counter-selection against *E. coli*. PCR analysis confirmed that the vector had integrated correctly into the chromosomal DNA. After sucrose treatment, transconjugants that were colistin resistant and chloramphenicol sensitive were selected, and the deletion was confirmed by colony PCR using A and D primers. Mutant validation was done by sequencing of AD fragments amplified from chloramphenicol sensitive mutants using hcp1Seq and vgrG1Seq primers (Table 2). The *A. hydrophila* mutants were designated *vAh*Δ*hcp1* and *vAh*Δ*vgrG1*.

### Virulence of vAh Mutants in Catfish Fingerlings

All fish experiments were conducted in accordance with a protocol approved by the Institutional Animal Care and Use Committee at Mississippi State University. Virulence of *vAh*Δ*hcp1* and *vAh*Δ*vgrG1* was compared to *A. hydrophila* wild-type (WT) strain ML09-119 by immersion route of exposure as described (Abdelhamed et al., 2016). Briefly, 120 6-month-old specific-pathogen-free (SPF) channel catfish fingerlings (18.10 ± 0.56 cm, 50.90± 3.76 g) were stocked into twelve 40-liter flow-through tanks (10 fish/tank) and acclimated for a week. Tanks were assigned randomly to four treatment groups: *vAh*Δ*hcp1, vAh*Δ*vgrG1*, vAh WT, and BHI (sham). Each group included three replicate tanks. Water temperature was maintained at 32°C (±2) throughout the experiments. Fish were fed twice a day with a commercial catfish feed. On the challenge day, the water levels in each tank were decreased to 10 L, and 100 mL of overnight culture was added directly to each tank (1.02 × 10^10^ CFU/mL water). Negative control tanks were exposed to 100 mL of sterile BHI broth. During immersion, water was well aerated. After 6 h, water flow was restored, and fish were maintained as usual. Fish mortalities were recorded daily for a total of 21 days, and percent mortality was calculated for each group. Protection against vAh WT challenge was determined in fingerlings that survived infection by the *vAh*Δ*hcp1* and *vAh*Δ*vgrG1* mutants. Briefly, at 21 days post-infection, catfish fingerlings were re-challenged by vAh WT by immersion (2.21 × 10^10^ CFU/ml water), and mortalities were recorded daily for 14 days. At the end of the experiments, mean percent survival was calculated for each treatment.

### Statistical Analysis

Mean percent mortality data were arcsine transformed, and analysis of variance (ANOVA) was applied using PROC GLM in SAS for Windows v9.4 (SAS Institute, Inc., Cary, NC) to assess significance. An alpha level of 0.05 was used in all analyses.

### Host-Pathogen Interaction Network

Protein-protein interactions between *A. hydrophila* secretion system proteins and catfish proteins (accession: PRJNA281269) were determined using the Host-Pathogen Interaction Database (Ammari et al., 2016). For pathogen sequences, default upload options were: database search: bacterial pathogens, matrix: Blosum62, *E*-value: 0.00001, pathogen percent identity: 30, and query coverage filter: 50%. For host sequences, selected animal protein options were: for the database search matrix: Blosum62, *E*-value cutoff: 0.00001, percent identity and query coverage filter: 70% (Ammari et al., 2016).

## Results

### Genome Features

The 55 genome sequences included in the current study are *A. hydrophila* isolates from different geographical locations and hosts (Table 1). Of these, our group sequenced vAh strains ML09-119, ML09-121, ML09-122, AL09-79, AL10-121, and Arkansas 2010. We also sequenced *A. hydrophila* strains AL06-06, AL97-91, MN98-04, and TN97-08. Additionally, we assembled and annotated 12 draft vAh strain genome sequences released in 2016 (strains Ahy_Idx71, ALG15-098, IPRS15-28, ML10-51K, S13-612, S13-700, S14-296, S14-458, S14-606, S15-130, S15-400, and S15-591) and one non-vAh strain genome (S14-230) for inclusion in our analysis. Genome size of the 55 strains ranged from ~4.67 to 5.28 Mb, and G+C ratio of the genomes ranged from 60.47 to 61.60.

### Average Nucleotide Identities (ANI) and Phylogenetic Tree Creation

**Figure 1:**
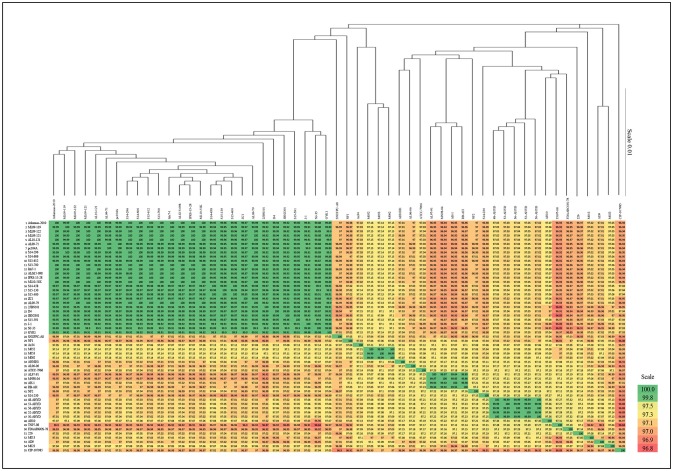
Average nucleotide identities (ANI) of *A. hydrophila* genomes and phylogenetic tree based on core genome. Note that branch lengths of the phylogenetic tree were reduced to fit the image.

A phylogenetic tree based on the complete core genome of 55 *A. hydrophila* strains shows the 27 vAh strains forming a highly conserved branch separated clearly from the other strains. The separation of the vAh cluster from the rest of the tree showed 100% branch conservation. These findings were confirmed by ANI as well as Average AAI (Supplementary File 1). ANI and AAI values within the cluster of 27 strains were above 99.88% (ANI) and 99.89% (AAI), respectively (Figure 1).

### Secretion Systems in *Aeromonas hydrophila* Genomes

**Figure 2:**
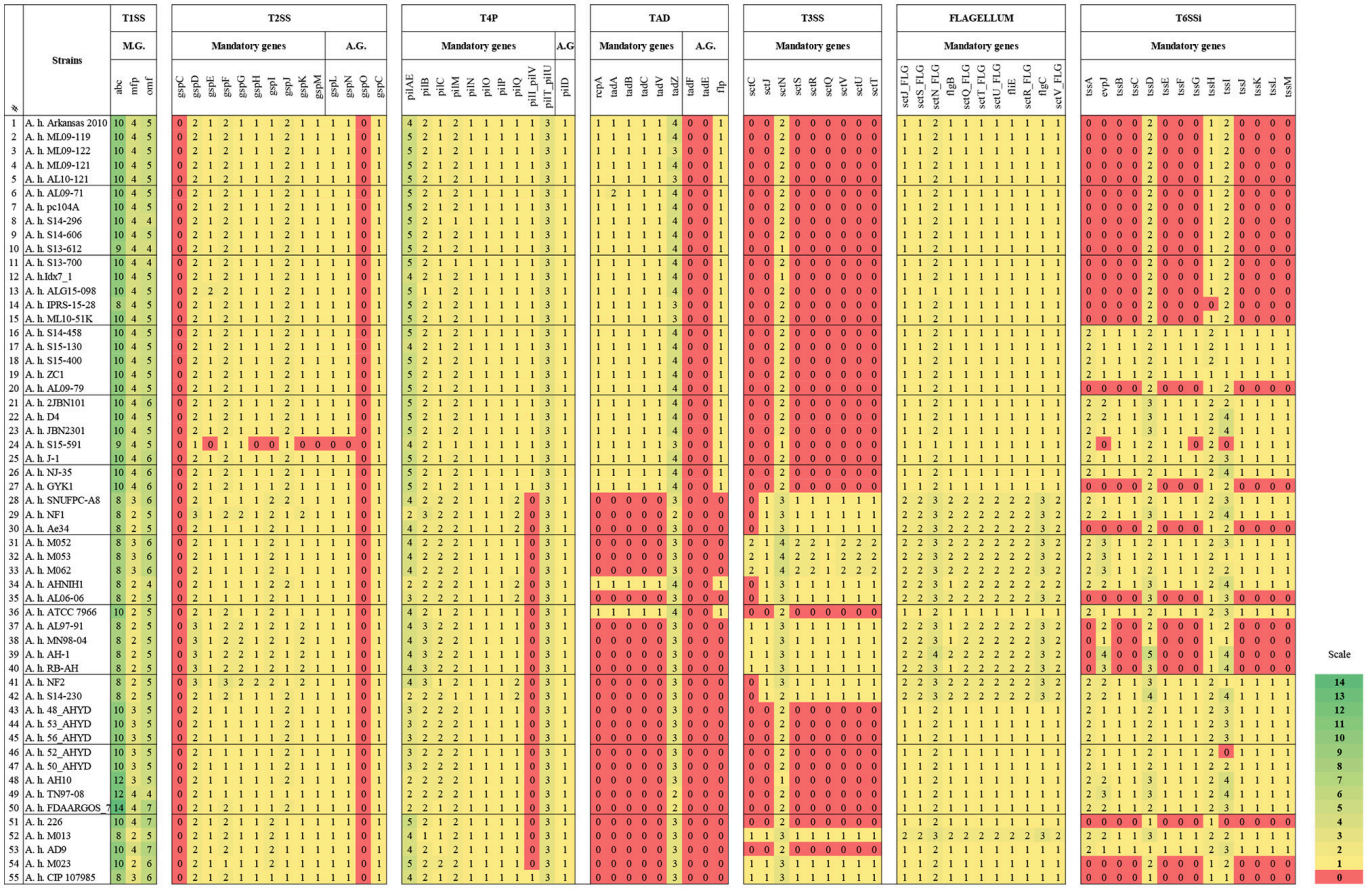
Core and accessory proteins of secretion systems, T4P, Tad, and flagella encoded in *A. hydrophila* genomes. Numbers and color represent the number of copies of each listed gene. The strains are listed in the same order as Table 1 and Figure 1 (the first 27 strains are vAh strains). *tssD* is also known as *hcp* and *tssI* is also known as *vgrG*. A.G. indicates Accessory genes.

In our *in silico* secretion systems analysis, we identified that most of the U.S.A. and Chinese vAh isolates tend to encode more T1SS core components, for instance ATP-binding cassette (*abc*) and *mfp* genes, compared to environmental isolates. Additionally, the genome of human strain FDAARGOS_78 encodes more *abc* and outer membrane factor (*omf*) genes than the other 54 genomes (Figure 2).

All of the evaluated *A. hydrophila* genomes encode a T2SS system except one: strain S15-591. However, this strain does not have a completed genome sequence; it is a draft assembly with a large number of contigs. Therefore, it is possible that the genes may not have been detected due to the large number of gaps in the genome (Figure 2).

All the vAh strains in the current study encode mandatory and accessory genes of type 4 pilus (T4P). By contrast, non-vAh strains from different origins and locations lacked the *pilQ* gene. One non-vAh strain (Ranae CIP 107985, which was isolated from a frog) encodes all the T4P elements (Figure 2).

Intriguingly, only one gene (*tadZ*) from the Tad system is present in all the evaluated *A. hydrophila* genomes. On the other hand, all the vAh strains as well as two non-vAh strains (ATCC 7966 and AHNIH1) encode this system (Figure 2).

The majority of vAh strains from the U.S.A. (except strain S14-230) and Chinese isolates do not carry all of the mandatory T3SS genes in their genomes (Figure 2). The mandatory T3SS gene cluster is composed of *sctU, sctJ, sctN, sctS, sctR, sctQ, sctV, sctU*, and *sctT*. Interestingly, only the *sctN* gene is present in all of the evaluated *A. hydrophila* genomes. Only two of the eight human isolates encode T3SS except for the *sctC* gene. *A. hydrophila* ATCC 7966 does not encode T3SS, but it has two copies of the *sctN* gene. By contrast, most of the environmental isolates carry T3SS. Interestingly, *A. hydrophila* strains that encode more T1SS components tend to have fewer or no genes encoding T3SS (Figure 2).

Some T3SS genes are similar to flagella genes. Therefore, we used MacSyfinder to discriminate between T3SS and flagella genes. Of the 55 *A. hydrophila* genomes we evaluated, all carry the mandatory flagella genes (Figure 2).

All of the evaluated *A. hydrophila* genomes encode either the entire operon or remnants of the T6SSi. Most of the U.S.A. vAh isolates have only three T6SSi genes: *hcp1 tssH*, and *vgrG*. By contrast, almost all the China isolates encode the entire T6SSi. The exception was strain GYK1 from China, which has the same three T6SSi genes as the U.S.A. vAh isolates. Additionally, fish isolate Ae34 from Sri Lanka, four non-vAh isolates from the U.S.A. (AL06-06, MN98-04, AL97-91, and Ranae CIP 107985), and one fish isolate (AH-1) from Canada do not encode the entire T6SSi elements but have the same three genes as the U.S.A. vAh strains. Three of the Malaysian isolates (M023, RB-AH, and 226) encode the same three elements from T6SSi, whereas four Malaysian isolates (M013, M052, M053, and M054) encode the entire T6SSi.

Only one gene encoding a T9SS-like protein (s*prA*) was identified in the *A. hydrophila* genomes. This gene is encoded by all the evaluated *A. hydrophila* genomes.

### Construction and Virulence of Mutant Strains

In the present study, we successfully introduced in-frame deletions in two genes encoding T6SS effectors: *hcp1* and *vgrG1* (Table 4). The Δ*hcp1* mutation has a deletion of 537 bp out of 564 bp (95.21%), and Δ*vgrG1* has a deletion of 2,739 bp out of 2,781 bp (98.49%).

**Table 4:** The sizes of upstream (USF), downstream (DSF), and in-frame fused fragments (FF), deleted region (DR), and undeleted region (UD) by base pair (bp).

**Gene name**	**Gene symbols**	**Locus tag**	**New locus tag**	**USF (AB)**	**DSF (CD)**	**FF (AD)**	**DR**	**UD**
Hcp-like protein	*hcp1*(*tssD*)	AHML_05970	AHML_RS05995	1,074	957	2,031	537	27
Rhs element Vgr protein	*vgrG1* (*tssI*)	AHML_05975	AHML_RS06000	990	1,038	2,028	2,739	42

**Figure 3:**
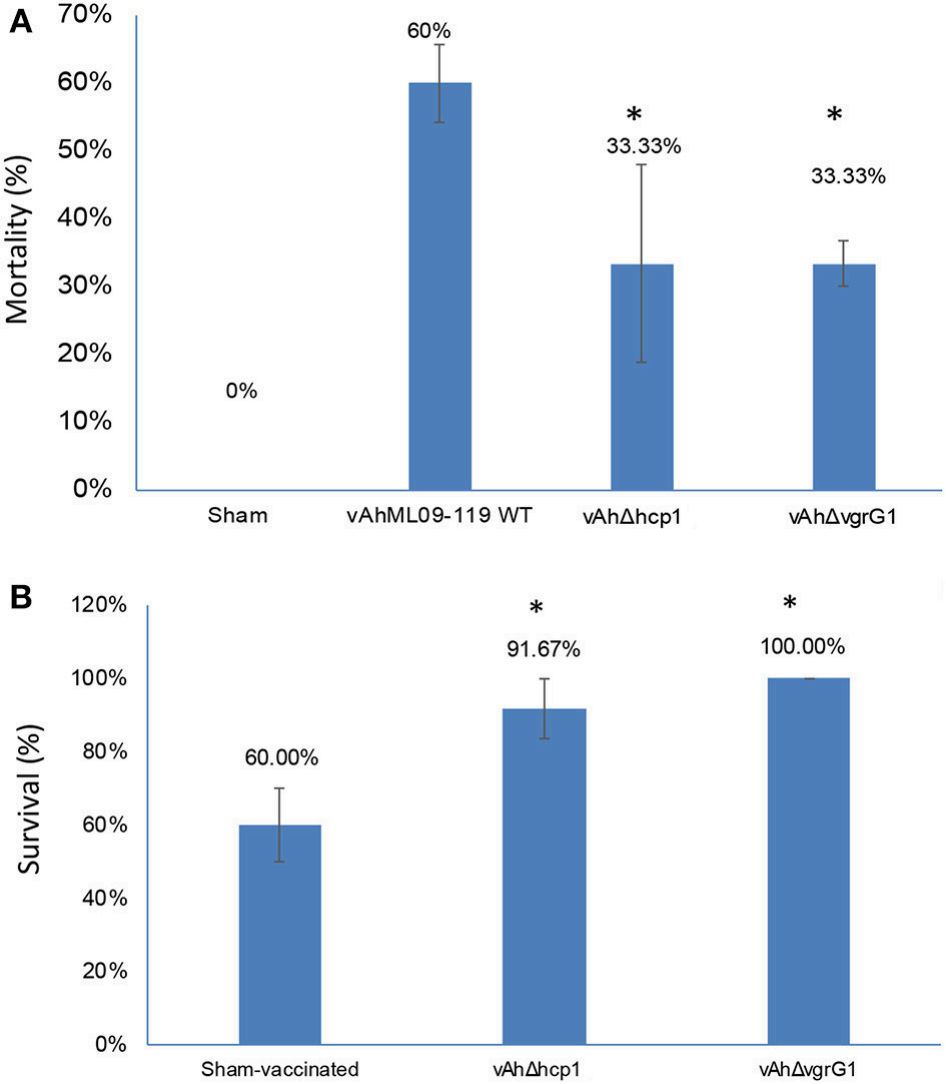
Virulence of vAh T6SS mutants in channel catfish fingerlings. **(A)** Percent mortalities in catfish fingerlings experimentally infected with vAh T6SS mutants and vAh wild type (WT) strain ML09-119. **(B)** Percent survival in catfish fingerlings surviving infection with T6SS mutants and re-challenged with vAh WT at 21 d post-infection. Data are the mean ± SE of three replicate tanks. Significant differences between challenged and non-vaccinated treatments are indicated with asterisks (*p* < 0.05).

Results of the immersion challenge in catfish fingerlings indicated that the mortality rate was significantly lower (*p* < 0.05) in *vAh*Δ*hcp1* and *vAh*Δ*vgrG1* compared with parent vAh strain ML09-119 (33.33 and 33.33% mortality vs. 60% mortality) (Figure 3A). Fingerlings surviving infection with *vAh*Δ*hcp1* and *vAh*Δ*vgrG1* had 91.67 and 100.00% percent survival, respectively, compared to 60.00% survival in the sham-infected control group (Figure 3B). In both experimental infections, all mortalities occurred within 72 h post-infection.

### Host-Pathogen Interaction

Using HPIDB, we predicted the interaction of identified *A. hydophila* secretion system components with host channel catfish (*Ictalurus punctatus*) proteins. We identified 333 catfish proteins that potentially interact with 30 different components of the *A. hydrophila* secretion systems (Supplementary File 2).

## Discussion

In this study, our goal was to compare the distribution of secretion systems in *A. hydrophila* genomes using comparative genomics. We found that some of the secretion systems commonly involved in pathogenesis of Gram-negative bacterial infections are not consistently present in the U.S.A. vAh isolates. However, there are three secretion systems (T1SS, T2SS, and T4P) present in all *A. hydrophila* strains we analyzed, and one system (Tad) that is present almost specifically in vAh strains. We determined that genes *hcp1 (tssD)* and *vgrG1 (tssI)* contribute to vAh virulence in catfish despite the absence of a complete T6SS.

Phylogenetic ANI analysis based on the complete core genome of the 55 strains in our study confirmed their classification as *A. hydrophila*, and it showed that the 27 vAh strains formed a highly conserved branch that is clearly separated from the other *A. hydrophila* strains. Also, ANI analysis showed that the U.S.A. vAh isolates and Chinese epidemic isolates were derived from the same monophyletic clade.

*Aeromonas hydrophila* secretes a wide range of extracellular enzymes and toxins. Type I secretion systems are capable of secreting exotoxins and enzymes by a one-step process from cytoplasm to outer membrane. T1SS consists of three main components: ATP-binding cassette (ABC) transporters, membrane fusion protein (MFP), and outer membrane factor (OMF) (Green and Mecsas, 2016). All of the evaluated *A. hydrophila* genomes carry core components of the T1SS. However, most of the vAh isolates and some of the other isolates encode additional copies of some genes encoding core components of the T1SS. A domain search analysis (data not shown) for all the evaluated 55 genomes revealed that the vast majority of the vAh isolates encode RTX toxins, which are cytotoxins that potentially cause host cell rounding and apoptotic death. In *Vibrio*, RTX toxin is secreted by T1SS (Boardman and Satchell, 2004). Presence of T1SS increases virulence of *Vibrio cholerae* (Dolores et al., 2015) and *Serratia marcescens* (Létoffé et al., 1996).

Not surprisingly, all the evaluated *A. hydrophila* genomes possess a T2SS. This system is capable of secreting enzymes such as proteases, phosphatases, and lipases (Korotkov et al., 2012; Green and Mecsas, 2016); in *A. hydrophila*, it is also well known for exporting cytotoxic enterotoxin (Act), which has hemolytic and cytotoxic activities (Chopra et al., 2000; Galindo et al., 2004; Korotkov et al., 2012). T2SS is a large, trans-envelope apparatus encoded by a set of 12-16 core genes. It is located in the outer membrane, and it transports folded proteins from periplasm into the extracellular environment. T2SS differs from T1SS, which releases proteins to the outer medium, and T3SS, T4SS, and T6SS, which are contact-dependent (Hayes et al., 2010). T2SS secretes specific toxins, effectors, and large proteins that could not be secreted to the host or competitor bacteria otherwise (Rondelet and Condemine, 2013; Rosenzweig and Chopra, 2013). T2SS has sequence similarity with the type 4 pilus (T4P) system, which is responsible for motility, signaling, and adhesion (Nivaskumar and Francetic, 2014). T4P has not been studied extensively in *A. hydrophila*. T4P and T2SS show a high degree of similarity in their components, and one of the genes encoding a T2SS component, *gspO*, is located in the T4P-encoding locus (Nivaskumar and Francetic, 2014). Our secretion system analysis assigned the *A. hydrophila gspO* gene as *pilD*, which is one of the accessory genes of T4P. *A. hydrophila gspC* gene is listed as a missing mandatory gene in Figure 2, but it is present as an accessory gene (Figure 2). In *E. coli*, T2SS and T4P are important for persistent infection (Kulkarni et al., 2009).

The tight adherence (Tad) system contributes to biofilm formation, colonization, and virulence of several pathogens (Tomich et al., 2007). The Tad system is similar to T2SS systems (Peabody et al., 2003; Tomich et al., 2007). Intriguingly, our results showed that a complete Tad secretion system is available in vAh strains, whereas the majority of the other evaluated *A. hydrophila* genomes do not encode a Tad system except for two strains, one of which is human clinical isolate (strain AHNIH1), and the other is from milk (strain ATCC 7966). Interestingly, only one gene (*tadZ*) from this system is available in all the evaluated *A. hydrophila* genomes. TadZ is encoded by one of the mandatory genes of the Tad system and plays a major role in mediating polar localization of the Tad secretion system (Perez-Cheeks et al., 2012).

Many Gram-negative pathogens use type III secretion systems, which delivers effector proteins directly into host cells. Many components of this system are homologous to flagellum proteins. T3SS is an important contributor to pathogenesis of some *A. hydrophila* strains (Vilches et al., 2004; Yu et al., 2004); however, our comparative genomics analysis showed that 27 vAh strains lack genes encoding T3SS (except for the *sctN* gene, which encodes a highly conserved ATPase that contributes to energy metabolism and provides recognition capability for T3SS effectors and other virulence factors) (Zarivach et al., 2007). Most of the non-vAh isolates in our study encode T3SS, but the majority of these are environmental isolates from outside the U.S.A. On the other hand, U.S.A. environmental isolates (soil and wetland sediment) do not encode a T3SS, and they also lack Tad systems. Our results showing absence of T3SS in vAh strains are consistent with a previous smaller-scale comparative genomics study (Pang et al., 2015). Therefore, presence of genes encoding a T3SS may not a good indicator of virulence potential for *A. hydrophila* strains in fish. Similarly, the majority of human clinical isolates (seven out of ten) do not encode T3SS.

Interestingly, there is an inverse relationship between presence of a Tad system and a T3SS in many of the *A. hydrophila* genomes we analyzed. The Tad system is encoded in the vAh isolates, but they do not encode T3SS. On the other hand, almost all of the non-vAh *A. hydrophila* strains do not encode a Tad system, but many of these genomes encode T3SS (Figure 2). Ten strains have neither Tad nor T3SS systems, and only one strain (human isolate AHNIH1) encodes both systems. Therefore, the three secretion systems consistently encoded in vAh strains are T2SS, T4P, and Tad.

Flagella are important in motility and often in attachment to the host. They are linked with biofilm formation, which contributes to persistent infection (Tomás, 2012). In eels, an *A. hydrophila* polar flagellum mutant had decreased survival and adherence to eel macrophages (Qin et al., 2014). Because flagella proteins are similar to T3SS proteins (Nguyen et al., 2000; Gophna et al., 2003), we included them in our comparative genomics analysis. All the evaluated *A. hydrophila* genomes encode mandatory flagella genes. In some bacteria, T3SS components play a role in flagellar rotation (Diepold and Armitage, 2015), but in *A. hydrophila*, there is only one T3SS gene (*sctN*) shared by all the evaluated *A. hydrophila* genomes. In *Sodalis glossinidius*, SctN mediated entry into tsetse fly cells (Dale et al., 2001). An *A. hydrophila* master regulator of T3SS (ExsA) negatively affects the lateral flagella (Zhao and Shaw, 2016), so it is possible that T3SS and flagella proteins interact in *A. hydrophila* strains encoding both systems.

T6SS is widely distributed in Gram-negative bacteria, and it contributes to bacterial fitness in specific niches (Cianfanelli et al., 2016). In particular, it delivers secreted proteins into competitor bacteria or host cells (Zoued et al., 2014). T6SS is categorized into three phylogenetic subtypes (T6SSi, T6SSii, T6SSiii) (Russell et al., 2014). All of the *A. hydrophila* genomes we evaluated encode the entire T6SSi operon or remnants of the T6SSi. Some of the U.S.A. vAh strains have only three genes (*hcp, tssH*, and *vgrG*) of T6SS, while other vAh strains from U.S.A. and China encode all the core genes of T6SS. We extended our research to understand the role of these remnants in the pathogenicity of *A. hydrophila*.

Strain ML09-119 encodes two hemolysin co-regulated proteins (Hcp) (AHML_05970 and AHML_10025) and two valine-glycine repeat G (VgrG) proteins (AHML_05975 and AHML_10030). The *hcp* genes are located adjacent to the v*grG* genes in strain ML09-119; the *hcp1* gene is adjacent to *vgrG1* gene, and *hcp2* gene is adjacent to the *vgrG2* gene. Multiple copies of *hcp* and v*grG* genes are commonly seen in several bacterial species that possess a T6SS, including *V. cholerae, Pseudomonas aeruginosa, A. hydrophila* SSU, and *A. hydrophila* ATCC 7966T (Mougous et al., 2006; Podladchikova et al., 2011; Sha et al., 2013).

Hcp and VgrG are effector proteins of T6SS (Cascales, 2008). However, structural analysis of Hcp and VgrG from *P. aeruginosa* and *V. cholerae* showed that these proteins independently formed a transportation channel between the inner and outer membranes through which other effector molecules can be transported to the host cell (Leiman et al., 2009; Pell et al., 2009). Thus, Hcp and VgrG could also be part of the secretion apparatus. Hcp and VgrG contribute to pathogenesis of several Gram-negative species, including *E. coli* (Dudley et al., 2006), *P. aeruginosa* (Hood et al., 2010), *Edwardsiella tarda* (Rao et al., 2004), and *Aeromonas* (Sha et al., 2013). In *V. cholera*, an *hcp1*/*hcp2* mutant is avirulent, whereas individual *hcp1* or *hcp2* mutants retain virulence. Therefore, at least one Hcp protein is required and sufficient for virulence (Pukatzki et al., 2006).

Secretion systems and effector proteins of *A. hydrophila* strain SSU have been studied extensively. It has been proposed that strain SSU be reclassified as *Aeromonas dhakensis* (Beaz-Hidalgo et al., 2015), but being a closely related species to *A. hydrophila*, SSU provides valuable comparative information. Strain SSU encodes a full T6SS, and its components are capable of translocating effector protein Hcp into eukaryotic cells (Suarez et al., 2008). Hcp modulates the activation of macrophages during infection in a mouse model (Suarez et al., 2010b). Effector protein VgrG is responsible for inducing host cell toxicity by ADP ribosylation of actin (Suarez et al., 2010a). In an intraperitoneal murine model of infection, all Hcp and VgrG paralogues were required for optimal *A. hydrophila* SSU virulence and dissemination to mouse peripheral organs (Sha et al., 2013).

vAh strain NJ-35 also encodes a functional T6SS that is located on a genomic island (Pang et al., 2015). This strain encodes three Hcp proteins. Hcp1 is responsible for T6SS assembly and inhibiting bacterial competition, Hcp2 negatively impacts biofilm formation and bacterial adhesion, and Hcp3 positively contributes to bacterial adhesion and biofilm formation (Wang et al., 2018). In NJ-35, all three genes contribute significantly to virulence, but a *hcp2* mutant had greater attenuation than *hcp1* and *hcp3* mutants (7-fold increase in LD_50_ for *hcp2* compared to 2-fold increase in LD_50_ for *hcp1* and *hcp3*).

In our study, deletion of the *hcp1* and *vgrG1* genes in vAh strain ML09-119 affected virulence significantly (Figure 3A). This finding is consistent with those reported for *A. hydrophila* strain SSU and vAh strain NJ-35, but both of these strains encode a functional T6SS, while strain ML09-119 does not. So what is the role of Hcp and VrgG in vAh strains that do not encode a functional T6SS? Our virulence data substantiates they could have similar roles in pathogenesis as Hcp and VgrG proteins in strains SSU and NJ-35. However, there is another intriguing possibility. It has been hypothesized that putative effector islands could be translocated by Hcp and VgrG (De Maayer et al., 2011), and it is worth noting that T6SS is encoded on a genomic island in vAh strain NJ-35. Therefore, it is possible that Hcp and VgrG mobilize effector islands in *A. hydrophila* and are responsible for the genomic variation in T6SS encoded in the species.

With the goal of developing an effective vaccine to protect catfish from MAS caused by vAh, we determined the level of protection provided by the Δ*hcp1* and Δ*vgrG1* mutants. Both mutants provided significant protection. The Δ*hcp1* and Δ*vgrG1* mutants are not safe enough for use as vaccines, but our results validate our approach of using comparative genomics to identify candidate virulence genes. Our results also indicate that deletion of virulence genes is a valid approach for live attenuated vaccine development against vAh.

T9SS is typically only found in some species in the Bacteroidetes phylum, so it is not surprising that only one gene (s*prA*) encoding a protein similar to T9SS is present in all the evaluated *A. hydrophila* genomes. T9SS functions as a secretion system but also enables gliding motility (McBride and Zhu, 2013; Sato et al., 2013; McBride and Nakane, 2015). In *Flavobacterium johnsoniae*, SprA is responsible (along with SprE and SprT) for secretion of SprB (Shrivastava et al., 2013).

Due to their role in secreting proteins involved in pathogenesis of multiple bacterial species, it is not surprising that 30 of the *A. hydrophila* secretion system proteins have predicted interactions with channel catfish proteins. We chose channel catfish as the host species for this analysis because of its importance as an aquaculture species in the U.S.A. and due to the impacts and known virulence of vAh strains on this species. These results confirm the multiple interactions between *A. hydrophila* secretion systems and channel catfish, adding additional evidence to their potential roles in *A. hydrophila* virulence.

In summary, our analysis indicates that vAh strains do not encode two of the contact-dependent secretion systems commonly involved in virulence of many Gram-negative pathogens, T3SS and T6SS. In fact, the T3SS is missing in all vAh strains and many other *A. hydrophila* strains. This suggests that vAh utilizes other systems to secrete effectors, toxins, and large secreted proteins. T1SS, T2SS, and T4P systems are encoded in all the *A. hydrophila* strains we sequenced, and these systems likely secrete several virulence-related proteins. Interestingly, the Tad system is present in all the vAh strains we sequenced, but it is only present in two of the non-vAh strains we analyzed. It is possible that the Tad system is one of the vAh-specific adaptations that make this clade of *A. hydrophila* more virulent.

Although only some *A. hydrophila* have a complete T6SS, all of the strains in our analysis encode three T6SS proteins. We determined that two of these genes, Δ*hcp1* and Δ*vgrG1*, contribute significantly to channel catfish virulence. Further investigation of the role of these T6SS genes in *A. hydrophila* is warranted, including the effects of deleting all the *hcp* and *vrgG* alleles on *A. hydrophila* virulence.

## Author Contributions

HT, HA, AK, and ML designed and conceived the analysis and experiments. HT, HA, JB, and SK performed experiments and analyzed the data. HT, HA, AK, and ML wrote the manuscript. All authors read and approved the final manuscript.

### Conflict of Interest Statement

The authors declare that the research was conducted in the absence of any commercial or financial relationships that could be construed as a potential conflict of interest.

## Supplementary Material

Supplementary File 1 AAI distances.

Supplementary File 2 Host-Pathogen interactions.
